# Evaluation of the effect of enzymatic pretreatment with l‐asparaginase on acrylamide formation during microwave, air, and deep frying of potatoes

**DOI:** 10.1111/1750-3841.70081

**Published:** 2025-03-20

**Authors:** Mariarca Esposito, Lucia De Luca, Giovanni Sorrentino, Giulia Basile, Martina Calabrese, Fabiana Pizzolongo, Raffaele Romano

**Affiliations:** ^1^ Department of Agricultural Sciences University of Naples Federico II, Piazza Carlo di Borbone I Portici Naples Italy

**Keywords:** acrylamide mitigation, cooking methods, Maillard reaction

## Abstract

**Summary:**

This study investigates the combined effects of l‐asparaginase pretreatment enzyme (10,000 ASNU/L) with three different frying techniques: deep, microwave, and air frying on acrylamide (AA) formation in French fries. Acrylamide content was analyzed using ultra‐high performance liquid chromatography coupled with a Diode Array Detector The study reveals that air‐fried potatoes contained the highest AA content among the three frying methods without enzyme pretreatment (963.83 µg/kg). In contrast, microwave‐fried potatoes had the lowest (785.31 µg/kg). The enzymatic pretreatment significantly reduced AA levels particularly in microwave‐fried potatoes, which recorded 482.28 µg/kg and 475.60 µg/kg after 20 and 30 min of enzyme treatment, respectively. These levels are below the reference threshold of 500 µg/kg as defined by Regulation (EU) 2158/2017. This study demonstrates that combining l‐asparaginase treatment with microwave frying can effectively mitigate AA levels in French fries by providing a healthier alternative to conventional frying methods.

**Practical Application:**

This study highlights the possibility of developing a safer fried potato products through the combined use of l‐asparaginase and microwave frying, effectively by reducing acrylamide levels below regulatory limits effectively. This strategy allows the optimization of frying processes, preserving the sensory quality of food and improving its safety. Furthermore, the proposed approach can be applied at both industrial, by offering innovative solutions for safer food preparation. Companies that adopt these techniques can benefit from regulatory compliance and increase the consumer confidence.

## 1 INTRODUCTION

Acrylamide (AA) is a potential human carcinogen formed in carbohydrate‐rich foods subjected to high temperatures (Truong et al., 2014). This reaction between reducing sugars, such as glucose and fructose and amino acids (in particular l‐asparagine), leads to the formation of *N*‐glycosides, which subsequently undergo Amadori rearrangement by forming AA as a result (Lineback et al., 2012). Due to its carcinogenic properties, AA has raised significant health concerns because of its presence being particularly prominent in fried potato products like French fries (da Cunha et al., 2019; EFSA Panel on Contaminants in the Food Chain (CONTAM), 2015). The widespread consumption of fried potato products in industrialized countries highlights the urgent need for strategies to reduce AA formation while preserving the sensory qualities of the food (Miranda & Aguilera, 2006; Pelucchi et al., 2003). Several approaches have been explored, including food additives, temperature adjustments, and enzymatic treatments. Among these, l‐asparaginase (EC 3.5.1.1) has emerged as a promising method, capable of hydrolyzing l‐asparagine into l‐aspartic acid and ammonia, thereby reducing the precursors necessary for AA formation (Hendriksen et al., 2009; Jia et al., 2021). Recent studies have highlighted the efficacy of l‐asparaginase in reducing AA levels in various food products, including French fries (Ciesarová & Kukurová, 2024). However, while there have been numerous studies on the individual effects of deep frying (DF), air frying (AF), and microwave frying (MF) on physico‐chemical and sensory characteristics (Giovanelli et al., 2017) and on AA formation, there is a notable gap in the literature regarding the comparative analysis of these frying methods when combined with l‐asparaginase pretreatment. Microwave frying has shown variable effects on AA content, with some studies reporting higher AA levels due to increased microwave power, while others suggest lower AA levels due to shorter frying times (Belgin Erdoǧdu et al., 2007; Mohd Ghazali et al., 2018). Air frying, a relatively new technique, offers a healthier alternative to DF by using hot air circulation, but its impact on AA formation was studied (Ahmed et al., 2023; Navruz‐Varlı & Mortaş, 2024), but the effect of enzymatic treatment combined with different types of frying was not studied on the best of our knowledge.

To address these gaps, this study aims to evaluate the effects of l‐asparaginase pretreatment on AA formation in French fries using three frying methods: DF, MF, and AF. By comparing these methods in a controlled setting, this research seeks to identify the most effective strategy for reducing AA content while maintaining the sensory qualities of French fries.

## 2 MATERIALS AND METHODS

Fresh potatoes (*Solanum tuberosum*, Colomba variety) and high‐oleic sunflowers oil (Friol, Ravenna) were purchased from a local market. The Colomba variety was selected because it belongs to one of the representative potato varieties for both the current fresh consumption market and the frying industry in Europe and also has a reducing sugar content of less than 0.30%, which is considered the threshold for a variety suitable for frying (Sowokinos, 2001).

All solvents and reagents used for the experiments were purchased from Sigma‐Aldrich Co. l‐Asparaginase obtained from *Aspergillus oryzae* (Acrylaway®) was supplied by Novozymes A/S. This enzyme has a molecular weight of approximately 36 KDa, with an optimal temperature of 60°C and pH 7.0.

### 2.1 Potato preparation

The potatoes were peeled with a knife. A potato stick cutter was used to cut the potatoes into strips with cross‐sections of 1 × 1 cm. A ruler and knife were then used to cut the strips to a length of 5 cm.

### 2.2 Pretreatments

Immediately after cutting, the potato strips were rinsed in distilled water for 1 min to remove surface starch. The cut potatoes were divided into three groups of 150 g each, which were then treated with a solution of l‐asparaginase (10,000 ASNU/L) at a 1:2 (w/v) potato‐to‐enzyme solution ratio at 40°C for 10, 20, and 30 min (coded as PT10, PT20, and PT30, respectively). According to the supplier's information, the optimal pH for enzyme is 6.5. Control sample (CP) of raw potato sticks rinsed in distilled water without enzyme treatment was also prepared.

### 2.3 Frying conditions

Three alternative procedures were used for frying approximately 50 g of untreated and enzymatically prepared potato sticks:

1. DF: Oil was added to the potato samples to achieve a potato‐to‐oil ratio of 1:10 (w/v). The samples were fried in an electric fryer (PETIT FRY 1200 W mod. 832010, DICTROLUX) at 170°C for 5 min.
2. AF: Oil was sprayed to the potato samples to achieve a 50:1 (w/v) potato‐to‐oil ratio. The samples were fried in an air fryer (DE LONGHI FH2101 1400 W) at 170°C for 10 min, turning the sample upside down after 5 min.
3. MF: Oil was added to the potato samples in a glass beaker to achieve a potato‐to‐oil ratio of 1:10 (w/v). The samples were fried in a microwave oven (SAMSUNG GE71A) at a power of 450 W for 3 min.

Before frying, the potatoes were dried with absorbent paper to remove surface water. Oil temperature was monitored during frying with a digital thermometer. After frying, the samples were cooled, frozen, and then freeze‐dried using a freeze dryer (LIO‐5PDGT, Cinquepascal S.R.L.). The freeze‐dried product was then ground with a laboratory mixer (WARING LB20EG) before the analysis. Table 1 shows the identification codes for each sample.

**TABLE 1:** Code of sample used for the study.

Enzyme treatment time (min)	Raw material	Deep frying	Microwave frying	Air frying
0	CP	DFC	MFC	AFC
10	PT10	DFT10	MFT10	AFT10
20	PT20	DFT20	MFT20	AFT20
30	PT30	DFT30	MFT30	AFT30

### 2.4 pH determination

pH was determined for samples before frying using a solids pH meter (TESTO 206) after appropriate calibration.

### 2.5 Water activity determination

Water activity (a_w_) was measured on grinded samples before frying using the Steroglass AQUALAB 4TE (METER Group, Inc.) device, which was appropriately calibrated to the standards.

### 2.6 Determination of dry matter

The dry matter was determined on samples before and after frying. Potato grinded samples (5 g) were dried at 105°C in an oven to a constant weight, following the AACC Method 44–15.02 (AACC International, 2013), to determine moisture content.

### 2.7 Determination of reducing sugars

Reducing sugars were extracted using the method described by Liyanage et al. (2021), with modifications. Briefly, 0.5 g of lyophilized sample was mixed with 10 mL of Milli‐Q water, 0.5 mL of Carrez I, and 0.5 mL of Carrez II were added to precipitate interfering substances. The samples were placed in an ultrasonic bath for 1 h and then centrifuged at 6500 rpm for 10 min. The supernatant was filtered, diluted 1:50 with Milli‐Q water, and analyzed. Monosaccharide content (d‐glucose and d‐fructose) in untreated and enzymatically pretreated samples before and after frying was determined spectrophotometrically using a Megazyme kit (d‐fructose/d‐glucose Kit, Megazyme®) according to the manufacturer's protocol. The wavelength was set at 340 nm. The total amount of reducing sugars was calculated as the sum of d‐glucose and d‐fructose and expressed in milligrams per 100 g dry weight.

### 2.8 Determination of l‐asparagine concentration

l‐Asparagine was extracted following the method described by Bachir et al. (2023) with some modifications. Briefly, 1 g of lyophilized sample was mixed with 30 mL of Milli‐Q water and placed in ultrasonic bath for 50 min. Milli‐Q water was then added to bring the volume to 50 mL. The solution was filtered, and l‐asparagine content in untreated and enzymatically pretreated samples before and after frying was determined using an l‐asparagine/l‐glutamine (rapid) assay kit (Megazyme®) according to the manufacturer's instructions. The wavelength was set at 340 nm. l‐Asparagine content in samples was expressed as milligrams per 100 g dry weight.

### 2.9 Determination of AA content

#### 2.9.1 Extraction

Acrylamide extraction was performed according to Cerit and Demirkol (2021). Briefly, 2 g of sample was mixed with 5 mL of hexane, vortexed for 2 min and centrifuged at 6500 rpm for 10 min. The supernatant was treated with 20 mL of formic acid 10 mM, 0.5 mL of Carrez I, and 0.5 mL Carrez II to precipitate co‐extracted colloids. After vortexing for 1 min and sonicating for 2 h, the mixture was centrifuged at 6500 rpm for 20 min. The supernatant was filtered through a SPHEROS filter (PES 0.45 µm, *d* = 25 mm). The solid residue was re‐extracted twice with 5 mL of 10 mM formic acid solution. The extracts were stored at 4°C for subsequent ultra‐high performance liquid chromatography system (UHPLC) analysis.

#### 2.9.2 UHPLC analysis

Acrylamide content was determined according to Covino et al. (2023), with modifications. An UHPLC (Jasco LC‐4000), equipped with an MD‐4010 PDA detector, CO‐4061 oven, UHPLC semi‐micro pump PU‐4285 and a reversed‐phase C‐18 column (Nucleodur C18 Gravity, 3 µm, 150×3 mm) with a precolumn was used.

The detector wavelength was set at 210 nm. Acrylamide was identified and quantified by comparing the retention time (4.6 min) and area of the external standard (0.25–50 ppm; *R* ^2^ = 0.99) with those of the samples, using a water‐formic acid solution (99.9:0.1) as the mobile phase at a flow rate of 0.25 mL/min under isocratic condition. Limit of detection and limit of quantification were 80 and 150 ppb, respectively. Furthermore, the percentage of recovery was 93%. The results were expressed in µg/kg of French fries.

### 2.10 Colorimetric analysis

Colorimetric indices (L*, a*, and b*) were measured on the surface of fried samples with a portable digital colorimeter (WR10QC, Beley) and the CIELab coordinates (L*, a*, b*) were recorded. The L* coordinate indicates lightness, ranging from black (0) to white (100). The chromaticity coordinates a* and b* represent red (+) to green (−) and yellow (+) to blue (−), respectively.

### 2.11 Statistical analysis

All experiments and related analytical measurements were performed in triplicate, and data were expressed as mean ± standard deviation (SD). Means of each parameter were assessed by analysis of variance (ANOVA) using Tukey's post hoc test (*p* ≤ 0.05). Statistical analysis was performed using XL‐STAT software (Addinsoft).

## 3 RESULTS AND DISCUSSION

### 3.1 pH and water activity

pH and a_w_ are critical factors that influence the activity of l‐asparaginase from *Aspergillus oryzae*. Table 2 presents the pH and a_w_ of each sample. The pH values range from 5.92 in CP to 6.10 in PT30. l‐Asparaginase is most active at a neutral pH range and at temperatures up to 60°C, suitable for treating food ingredients or products (Hendriksen et al., 2009). According to the supplier's information, the optimal pH and temperature for Acrylaway® are 6.5 and 58°C, respectively (Rottmann et al., 2021). The pH value of the fresh potatoes in this study aligns with values reported by Amaral et al. (2015), who found a range between 5.97 and 5.38. However, an increase in pH was observed during enzymatic treatment, especially in PT30 compared to control. The a_w_ ranged from 0.98 in CP to 0.99 in PT30, consistent with the findings of Reis et al. (2008), who reported high a_w_ values (close to 1.00) in raw potatoes.

**TABLE 2:** pH and water activity (a_w_) of sample control and enzymatic pretreated potatoes (raw potatoes).

Sample	pH	a_w_
CP	5.92 ± 0.01^b^	0.981 ± 0.000^c^
PT10	6.03 ± 0.01^ab^	0.985 ± 0.001^b^
PT20	6.05 ± 0.02^ab^	0.988 ± 0.001^ab^
PT30	6.10 ± 0.06^a^	0.991 ± 0.002^a^

*Note*: ^a‐c^Different letters in the same column indicate statistically significant differences (*p* < 0.05).

### 3.2 Dry matter

Table 3 provides raw and cooked potatoes dry matter content. The dry matter content in raw potatoes ranged from 17.63 g/100 g in PT30 to 23.82 g/100 g in CP. These results are consistent with those reported by Islam et al. (2022), who found dry matter value in different potato varieties around 20 g/100 g. Potato tubers generally contain 70%–80% water (Reyniers et al., 2020). The treatment time reduce dry matter percentage, with the lowest content observed in PT30 (17.63 g/100 g). This decrease may result from water uptake during enzymatic treatment due to starch's water absorption capacity. When starch is added to water, water molecules penetrate the starch granules hydrating them (Zhang et al., 2024). Components such as starch and reducing sugars may have leached during soaking (Nematollahi et al., 2021).

**TABLE 3:** Dry matter (g/100 g) of sample control, enzymatic pretreated potatoes (raw potatoes) and French fries.

Raw potatoes	
Sample	Dry matter
CP	23.82 ± 1.43^a^
PT10	23.11 ± 1.24^a^
PT20	19.07 ± 1.87^a^
PT30	17.63 ± 3.26^a^

French fries	
Sample	Dry matter
DFC	38.24 ± 0.94^c^
DFT10	37.38 ± 0.62^c^
DFT20	32.96 ± 1.39^c^
DFT30	34.72 ± 0.60^c^
MFC	70.64 ± 1.31^a^
MFT10	61.98 ± 2.01^ab^
MFT20	57.78 ± 1.57^b^
MFT30	59.17 ± 0.61^b^
AFC	35.58 ± 2.49^c^
AFT10	30.89 ± 5.90^c^
AFT20	31.24 ± 3.53^c^
AFT30	33.09 ± 3.18^c^

*Note*: ^a‐c^Different letters in the same column indicate statistically significant differences (*p* < 0.05).

The dry matter content in cooked potatoes varied depending on the frying method. Specifically, in samples obtained by DF, the dry matter in the control and the samples enzymatically treated at different times were statistically distinct, ranging from 32.96 g/100 g (DFT20) to 38.24 g/100 g (DFC). Similar results were reported by Oztop et al. (2007), who found a dry matter content of 33 g/100 g after frying at approximately 170°C for 4 min in sunflower oil. The higher dry matter content in the samples analyzed here, compared to those reported in the study, may be due to the longer frying time (5 min) and, thus, more significant dehydration of the product.

In MF samples, the dry matter values ranged from 57.78 g/100 g (MFT20) to 60.64 g/100 g (MFC), consistent with data obtained by Ahmed et al. (2023).

Statistically significant differences were found between MFC, MFT20, and MFT30. The dry matter content in AF samples ranged from 30.89 g/100 g (AFT10) to 35.58 g/100 g (AFC), with no significant differences among samples. These results are consistent with those reported by Ahmed et al. (2023), who found a dry matter content of 35.60 g/100 g for samples obtained by AF at 170°C for 10 min.

Dry matter content is a crucial parameter in food products, as it directly affects texture, color, and sensory acceptability (Ahmed et al., 2023). During the production of French fries, thermal processes lead to a marked and irreversible change in the structure of the potato and a significant increase in dry matter, resulting in a change in the texture of the product (Lisińska & Gołubowska, 2005). Desirable sensory properties of French fries, including crispness, develop during frying and therefore, in addition to the quality of the raw potatoes, the conditions during frying will influence the quality of the final product (Albuquerque et al., 2012). The observed decrease in moisture in DF and MF compared to AF can be attributed to the distinct heat transfer mechanisms involved in each method. In DF, the samples were immersed in hot oil, which has a higher thermal conductivity than air, enabling faster heat transfer from the oil to the food surface and leading to a more rapid decrease in moisture content. Similarly, the differences in dry matter values observed in MF compared to air and DF were expected, as microwaves significantly enhance moisture losses (Oztop et al., 2007). Parikh and Takhar (2016) noted that microwaves promote water pumping from the interior to the surface of the food during cooking, resulting in a significantly higher dry matter content in fried potatoes compared to other methods, as also reported by Zhou et al. (2022). This can be attributed to the unique heating mechanism of microwaves, where the internal pressure within high‐moisture food rises rapidly under microwave heating, reaching levels higher than those generated by conventional methods. Under these high‐pressure conditions, moisture is forced toward the food's surface. However, the surface cannot effectively retain this moisture, causing it to be expelled in liquid form without undergoing a phase change, a phenomenon commonly referred to as “Liquid Pumping” (Ozkoc et al., 2014).

### 3.3 Reducing sugar and l‐asparagine content

Table 4 presents the reducing sugar and l‐asparagine content for each sample. Initially, the reducing sugar content in CP was 6.80 g/100 g of dry weight. After soaking, the reducing sugar content in potatoes decreased, with a reduction of 9.85% in PT30. This decrease, although not statistically significant, is likely due to the dissolution of sugars in water, as reported by Mestdagh et al. (2008). Dias et al. (2017) have described similar findings. In the cooked samples, the percentage of reducing sugars in French fries increased with the duration of l‐asparaginase treatment.

**TABLE 4:** Reducing sugar (g/Kg d.w.) and asparagine content (g/100 g d.w.) in sample control, enzymatic pretreated potatoes (raw potatoes) and French fries.

Raw potatoes		
Sample	Reducing sugar	Asparagine content
CP	6.80 ± 0.34^a^	2.04 ± 0.12^a^
PT10	6.64 ± 0.52^a^	1.97 ± 0.06^a^
PT20	6.31 ± 0.07^a^	1.57 ± 0.08^b^
PT30	6.13 ± 0.44^a^	1.22 ± 0.07^b^

French fries		
Sample	Reducing sugar	Asparagine content
DFC	5.31 ± 1.46^d^	1.42 ± 0.02^a^
DFT10	6.47 ± 1.20^bcd^	1.35 ± 0.02^ab^
DFT20	9.51 ± 0.19^abc^	1.16 ± 0.04^cd^
DFT30	9.81 ± 1.33^ab^	1.04 ± 0.03^d^
MFC	5.77 ± 0.43^cd^	1.43 ± 0.04^a^
MFT10	7.15 ± 0.23^bcd^	1.36 ± 0.01^ab^
MFT20	7.28 ± 0.50^bcd^	1.34 ± 0.03^ab^
MFT30	8.90 ± 0.99^abcd^	1.17 ± 0.04^c^
AFC	7.82 ± 0.04^abcd^	1.39 ± 0.01^a^
AFT10	8.51 ± 1.34^abcd^	1.32 ± 0.03^ab^
AFT20	9.25 ± 1.63^abc^	1.25 ± 0.04^bc^
AFT30	11.43 ± 0.52^a^	1.05 ± 0.04^d^

*Note*: ^a‐d^Different letters in the same column indicate statistically significant differences (*p* < 0.05).

Specifically, the content of reducing sugars in the treated samples increased by 45.87% in DFT30, 31.58% in AFT30, and 35.16% in MFT30 compared to control. Monitoring the reducing sugar content is essential as these sugars are key reactants in the Maillard reaction, directly affecting the sensory characteristics of the final product (Dias et al., 2017).

In all frying methods, the increase in reducing sugar content correlated with a decrease in l‐asparagine (Table 4) during the frying process, which intensified with longer enzyme pretreatment times, demonstrating the effectiveness of the enzymatic treatment. A progressive decrease in asparagine content was observed as contact time with the enzyme increased, with a reduction of up to 40% from CP to PT30. Similar results were obtained by Marquez and Añon (1986) who observed an increase in reducing sugars and a reduction in free amino acids following DF. The increase in reducing sugars observed in French fries, upon prolonged pretreatment with l‐asparaginase, could be attributed to a decreased availability of substrate (asparagine). However, this increase may also result from other metabolic pathways or carbohydrate degradation processes during heating. In particular, after frying, the increase in reducing sugars could be related to a process of starch hydrolysis, which occurs mainly in the potato flesh. This process would favor the formation of reducing sugars from complex carbohydrates in the pulp, mitigating the effect of the release of reducing sugars during soaking (Marquez & Añon, 1986). It is well established that the primary reactants in AA are sugars and the amino acid l‐asparagine (Taeymans et al., 2004). The enzyme asparaginase catalyzes the hydrolysis of asparagine into aspartic acid and ammonia, significantly reducing the level of free asparagine and, consequently, the amount of substrate available for the formation of AA (Zyzak et al., 2003). The relationship between l‐asparagine and reducing sugar concentrations in fresh potatoes and AA formation during processing is complex (Yang et al., 2016). Halford et al. (2012) suggested that AA formation during processing is proportional to sugar content when sugar concentration is high in raw potatoes. In contrast, at low sugar levels in raw potatoes, AA formation is proportional to l‐asparagine content.

Furthermore, Amrein et al. (2003) reported that the potato cultivars showed differences in the AA formation depending primarily by sugar contents, while the asparagine content is less influenced by cultivar. In potatoes, l‐asparagine is the most abundant free amino acid, sometimes accounting for more than 50% of all free amino acids (Dias et al., 2017). Therefore, monitoring l‐asparagine content is decisive in evaluating the effectiveness of enzyme treatment. Since asparagine concentrations are relatively lower than reducing sugars in potatoes, asparagine is the limiting factor for AA formation in fried products (Muttucumaru et al., 2017).

After frying (see Table 4), the l‐asparagine content reduced by 30.39% in DFC, 31.86% in AFC, and 25% in MFC compared to CP, indicating that the l‐asparagine content was consumed during the Maillard reaction (Dias et al., 2017). A higher l‐asparagine content was observed in the microwave‐fried (MFC) sample, but it was not statistically significant compared to DFC and AFC. This finding highlights the unique characteristics of MF and aligns with the observed reduction in AA content associated with this particular frying technique.

### 3.4 Acrylamide content

**FIGURE 1:**
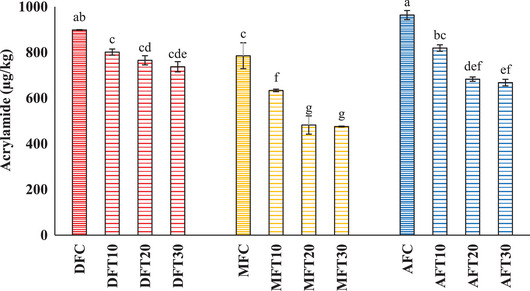
Acrylamide content in French fries. ^a‐g^Different letters in the same column indicate statistically significant differences (*p* < 0.05).

Figure 1 shows the AA content in French fries prepared using different frying methods and treatment times with l‐asparaginase. The results demonstrate significant variations in AA content depending on the frying method and the duration of enzyme treatment.

Among the frying methods examined, AF (AFC) produced the highest AA level at 963.81 µg/kg, while MF (MFC) yielded the lowest level at 785.34 µg/kg, consistent with findings by Ahmed et al. (2023). Compared to DF (DFC), which had an AA level of 898.59 µg/kg, MF resulted in a 12.60% decrease in AA, whereas AF caused a 7.26% increase, although not statistically significant.

The higher asparagine content in microwave‐fried potatoes, despite the lower formation of AA, suggests that factors beyond asparagine availability have influenced AA synthesis. The reduced AA content in microwave‐fried potatoes (MFC) could be explained by the particular heat transfer mechanism characteristic of this technology (Sansano et al., 2018). As previously reported, during MF, volumetric heating generates an internal steam pressure that facilitates the escape of moisture (Parikh & Takhar, 2016). This steam flow, which moves from the product surface to the surrounding oil, could contribute to the removal of some of the formed AA, a compound known for its instability and volatility (Amrein et al., 2006). Furthermore, the application of microwaves intensifies the volatilization of AA, due to the volumetric heating and increased moisture flow generated (Belgin Erdoǧdu et al., 2007).

As expected, the application of l‐asparaginase treatment prior to frying reduced AA levels significantly across all frying methods as the treatment time increased. Specifically, AA levels decreased by 18% in DFT20 and DFT30 compared to DFC, approximately 40% in MFT20 and MFT30 compared to MFC, and around 31% in AFT20 and AFT30 compared to AFC.

Notably, the AA content in MFT30 (475.61 µg/kg) and in MFT20 (482.18 µg/kg) complied with the reference level of less 500 µg/kg set by Regulation (EU) 2017/2158 (2017).

These results highlight the potential of combining enzymatic treatment with MF as an effective strategy to reduce AA levels in French fries. In addition, the lack of statistically significant differences between MFT20 and MFT30 suggests that a 20‐min enzymatic treatment might be sufficient for optimizing processing time without compromising the reduction in AA content.

### 3.5 Colorimetric analysis

Table 5 presents colorimetric parameters (L*, a*, b*) of French fries subjected to enzymatic treatment and different frying methods. The L* values, representing the darkness or lightness of color, ranged between 38.50 and 55.32 in fried potato, while the b* values, indicating the yellow direction, were above 10, and a* values, indicating the red direction, were between 1.05 and 9.58.

**TABLE 5:** Colorimetric parameters of French fries.

Sample	L*	a*	b*
DFC	42.75 ± 0.76^d^	9.58 ± 0.69^a^	24.38 ± 1.30^bc^
DFT10	44.58 ± 0.28^c^	7.63 ± 0.06^b^	21.30 ± 1.03^cd^
DFT20	48.41 ± 0.58^b^	4.28 ± 0.43^c^	18.16 ± 0.89^de^
DFT30	43.85 ± 0.55^cd^	2.05 ± 0.18^ef^	14.29 ± 0.59^fg^
MFC	38.50 ± 1.02^e^	8.66 ± 0.24^ab^	39.06 ± 2.80^a^
MFT10	44.31 ± 0.92^cd^	8.11 ± 0.94^b^	23.78 ± 1.66^bc^
MFT20	54.80 ± 0.47^a^	4.57 ± 0.44^c^	24.46 ± 0.62^bc^
MT30	49.06 ± 0.10^b^	3.59 ± 0.04^cd^	25.52 ± 0.02^b^
AFC	49.51 ± 0.13^b^	2.77 ± 0.12^de^	16.30 ± 0.03^ef^
AFT10	55.32 ± 0.86^a^	2.49 ± 0.32^de^	9.88 ± 0.48^h^
AFT20	54.01 ± 0.32^a^	2.25 ± 0.15^ef^	13.97 ± 0.39^fg^
AFT30	54.72 ± 0.52^a^	1.05 ± 0.03^f^	11.87 ± 1.97^gh^

*Note*: ^a‐h^Different letters in the same column indicate statistically significant differences (*p* < 0.05). L* indicates darkness or lightness of color. Coordinates a* and b* indicate color directions: +a* is the red direction, –a* is the green direction, +b* is the yellow direction, and –b* is the blue direction.

The L* index is a critical parameter in the frying industry, as it is generally the first attribute consumers perceive when evaluating product quality. Statistically significant increases in the L* index were observed in the enzymatically pretreated fried samples compared to the control. This increase in brightness (L*) is consistent with the reduction in AA levels, as lower AA content in French fries is directly related to higher L* values (Cerit & Demirkol, 2021).

Comparing the various frying techniques, the air‐fried product (AFC) exhibited a significantly lower a* parameter, resulting in a lighter color than the deep‐fried and microwave‐fried products. Verma et al. (2023) reported similar results. The lighter color of AFC could be attributed to the lower oil absorption during frying, as the final color of the product depends on both oil absorption and the chemical browning reactions of reducing sugars and protein sources (Oztop et al., 2007). Oil absorption plays a key role in the visual perception of color by altering the surface properties of fried products, which can affect light reflection and, consequently, apparent brightness. This was reported by Yang et al. (2016), who stated that the oil absorption and color parameter values have a strong correlation. Specifically, the H value, representing the perceived color hue (i.e., the position of the color in the a*‐b* chromatic plane), was found to decrease with increased oil absorption, resulting in a shift toward a more yellowish hue.

The a* parameter decreased significantly from the control to fried products after 30 min of enzyme treatment. The a* component is an indicator of nonenzymatic browning and positively correlated with AA content (Gikundi et al., 2024). Acrylamide such as melanoidins is a byproduct of the Maillard reaction in food processed at temperature > 120°C (Yang et al., 2016). The imparting of brown color is mainly attributed to melanoidins (Murata, 2021).

Color is an important factor influencing consumer acceptability, with a golden color indicating high product quality. Consumers generally use color to determine the end of the frying process (Oztop et al., 2007).

The color change generally occurs during the progress of the Maillard reaction. The initial pale color of the raw potato turns to a goldenbrown color due to the production of melanoidins, brown polymers formed through complex chemical browning processes between reducing sugars and amino acids (Ahmed et al., 2023). The color change during frying is correlated with a decrease in L* and/or an increase in the a* parameter (redness) (Genovese et al., 2019).

## 4 CONCLUSION

The results demonstrated that enzymatic treatment effectively reduced asparagine levels in the intermediate processing stages, leading to a corresponding decrease in AA content in the finished product. Variations in AA content were observed depending on the frying method used, with the lowest levels recorded in microwave‐fried products and the highest in air‐fried products. Notably, 30 and 20 min of enzymatic treatment with MF yielded the lowest AA levels in French fries. These findings suggest that such combinations could reduce AA levels without requiring specialized equipment or complex applications.

So, it is important to implement pretreatment and frying techniques to reduce AA levels in French fries and other food products while also maintain nutritional quality, safety and sensory attributes to ensure consumer acceptance.

## AUTHOR CONTRIBUTIONS

**Mariarca Esposito**: Formal analysis; writing—original draft; writing—review and editing. **Lucia De Luca**: Conceptualization; methodology; supervision; writing—review and editing; validation. **Giovanni Sorrentino**: Writing—original draft; data curation. **Giulia Basile**: Software; data curation; investigation. **Martina Calabrese**: Formal analysis; investigation. **Fabiana Pizzolongo**: Investigation. **Raffaele Romano**: Funding acquisition; conceptualization; project administration; methodology; supervision.

## CONFLICT OF INTEREST STATEMENT

The authors declare no conflicts of interest.
